# A spatio-temporal learning-based model for sleep apnea detection using single-lead ECG signals

**DOI:** 10.3389/fnins.2022.972581

**Published:** 2022-08-05

**Authors:** Junyang Chen, Mengqi Shen, Wenjun Ma, Weiping Zheng

**Affiliations:** ^1^School of Computer Science, South China Normal University, Guangzhou, China; ^2^Grado Department of Industrial and Systems Engineering, Virginia Polytechnic Institute and State University, Blacksburg, VA, United States

**Keywords:** sleep apnea, ECG signals, spatio-temporal learning, BiGRU, attention

## Abstract

Sleep apnea (SA) is a common chronic sleep breathing disorder, which would cause stroke, cognitive decline, cardiovascular disease, or even death. The SA symptoms often manifest as frequent breathing interruptions during sleep and most individuals with sleeping disorders are not aware of the SA events. Using a portable device with single-lead ECG signal is an effective way to help an individual to monitor their sleep conditions at home. However, the SA detection performance of ECG-based methods is still difficult to meet the clinical practice requirement. In this study, we propose an end-to-end spatio-temporal learning-based SA detection method, which consists of multiple spatio-temporal blocks. Each block has the identical architecture with a convolutional neural network (CNN) layer, a max-pooling layer, and a bi-gated recurrent unit (BiGRU) layer. This architecture with repeated spatio-temporal blocks can well capture the morphological spatial feature information as well as the temporal feature information from ECG signals. The proposed SA detection model was evaluated on the publicly available datasets of PhysioNet Apnea-ECG dataset (Apnea-ECG) and University College Dublin Sleep Apnea Database (UCDDB). Extensive experimental results show that our proposed SA model on both Apnea-ECG and UCDDB datasets achieves state-of-the-art results, which are obviously superior to existing ECG-based SA detection methods. It means that our proposed method has the potential to be deployed into a healthcare system to provide a sleep monitoring service, which can screen out SA population with high risk and help to take timely interventions to prevent serious consequences.

## 1. Introduction

Sleep apnea (SA) is a sleep disorder in which breathing is interrupted several times during sleeping. Its typical symptoms include headache, insomnia and others, and it can be potentially serious (Russell et al., 2014). Without prompt and appropriate treatment measures, patients with SA would suffer from serious complications such as stroke (King and Cuellar, 2016), cognitive decline (Vanek et al., 2020), cardiovascular disease (Lin et al., 2020), and even death. SA is considered by some researchers to be a recognized independent risk factor for stroke, such that individuals with SA have an approximately twofold greater risk of stroke compared with those without SA (Lyons and Ryan, 2015). This indicates that SA is a great threat to the global physical and mental health, with ~936 million adults (male and female) aged 30–69 years worldwide suffering from mild to severe obstructive sleep apnea, and 425 million adults aged 30–69 years suffering from moderate to severe obstructive sleep apnea (Benjafield et al., 2019). Due to the prevalence of SA, it is very vital to screen out individuals with SA and take timely interventions.

In clinical practice, polysomnography (PSG) is the gold standard test to diagnose SA. However, conducting PSG is expensive and often unavailable due to the shortage of physical therapists and sleep monitoring units (Graco et al., 2018). PSG with many biomedical sensors, including electroencephalogram (EEG), electro-oculogram (EOG), electromyogram (EMG), electrocardiogram (ECG) and pulse oximetry as well as airflow and respiratory effort, is performed as an SA test overnight in a sleep laboratory or specific unit in a hospital (Ali et al., 2019). This can be quite cumbersome and uncomfortable, so the collected signals cannot reflect the individuals' sleep conditions. In addition, a physical therapist must be available when the PSG is conducted in the hospital, which significantly restricts the screening of people with SA.

Home Sleep Test (HST) is an alternative to PSG for SA diagnosis, which is usually conducted overnight outside of the hospital or sleep laboratory (Rosen et al., 2017). Portable devices, which are simple, of low-cost and easy to operate, have been developed to enable the patients to monitor their sleep conditions in an unattended home environment. Gaiduk et al. (2020) have developed a system based on pressure sensors that can work independently and *via* wireless connection, which is as accurate as the current technology. However, this pressure sensor-based approach is highly sensitive to pressure, and the pressure signal can be easily contaminated with noise from the external environment. ECG is considered to be one of the most relevant physiological signals for the SA detection because patient's heart rate increases when SA occurs (Somers et al., 2008; Wang T. et al., 2019). ECG contains valuable information about the cardiorespiratory system and is therefore of great importance for SA detection (Bahrami and Forouzanfar, 2021). Over the past twenty years, various approaches have been proposed for the automated SA detection using HRV and EDR signals which can be derived from ECG (Gutiérrez-Tobal et al., 2015; Faust et al., 2016; Smruthy and Suchetha, 2017; Viswabhargav et al., 2019). In addition, ECG can be easily recorded. Therefore, ECG-based portable devices represent a better option. A wearable ECG acquisition system has been developed (Weder et al., 2015), which is designed as a chest strap that can continuously monitor ECG signals for multiple nights. As a more comfortable option, Ankhili et al. (2018) developed a reliable, washable ECG monitoring undergarment that can record and send the ECG signal wirelessly to a smartphone to analyze the ECG signal in real-time.

Using ECG signals can greatly reduce the complexity of diagnostic SA tests and allow for better monitoring of physiological changes in the patient (Bsoul et al., 2011). Several algorithms have been proposed for ECG-based SA detection. These algorithms generally include a first step of feature extraction from the original ECG signals, and then these features are used as the input and fed into a classification model (Baty et al., 2020). Sharma and Sharma (2016) extracted features from QRS complex waves using Hermite decomposition. Then, these features were combined with time series features, and least squares-support vector machine (LS-SVM) was used as a classifier for SA detection. Song et al. (2015) introduced a classifier that blends an SVM with a hidden Markov model (HMM) to take advantage of the time-dependent nature of SA segments. In recent years, deep neural networks (DNN) with end-to-end training are also applied to build SA detection models. Li et al. (2018) used HMM, ANN, and ECG signals for the identification of SA segments. Feng et al. (2020) used an unsupervised neural network to learn features, and they improved the performance of the classifier by taking into account the time-dependence and imbalance problems.

Although aforementioned models achieved promising results, there still exists a gap between their SA detection performance and the requirement of actual applications. Note that spatial patterns and temporal correlations are both important for SA detection. For example, the R-peak has salient spatial features on the ECG signals; while the RR intervals can be derived from the temporal dependencies. In reality, RR intervals are frequently utilized in SA detection (Bahrami and Forouzanfar, 2021). However, the spatio-temporal correlations are seldom utilized in the existing SA detection models (Sharan et al., 2020; Chen et al., 2021; Yang et al., 2022). Bahrami and Forouzanfar (2021) has used a hybrid CNN and LSTM network to extract spatio-temporal feature from ECG signals. However, they only use a simple combination of CNN and LSTM networks. To improve the performance of SA detection, a spatio-temporal learning based DNN model is proposed in this paper. In order to take advantage of more spatio-temporal dependencies, multiple adjacent segments are concatenated and used as the input of the proposed model. A spatio-temporal learning block is designed which is composed of a one-dimensional convolutional neural network (CNN), a max-pooling layer, and a bidirectional gated recurrent unit (BiGRU). Multiple spatio-temporal blocks are stacked in the proposed model to extract long-range spatial and temporal correlations. As a result, this model can fully utilize the multiple concatenated ECG segments. Moreover, an attention mechanism is employed to further utilize the global correlations by using the high-level forward and backward spatio-temporal features. These characteristics have made our model different with the other spatio-temporal model (Bahrami and Forouzanfar, 2021). The performance of our model has outperformed the one of Bahrami and Forouzanfar (2021). Experimental results on two public domain datasets of PhysioNet Apnea-ECG dataset (Apnea-ECG) and University College Dublin Sleep Apnea Database (UCDDB) showed that CNN-BiGRU achieved the competitive performance to the previous state-of-the-art methods. The main contributions of this study can be listed as follows:

- To fully extract spatio-temporal information from ECG signals, we proposed a spatio-temporal learning-based model called CNN-BiGRU with multiple spatio-temporal blocks, each block of which consists of a one-dimensional CNN layer, a max-pooling layer, and a BiGRU layer.
- We employed an attention mechanism to further exploit the high-level forward and backward spatio-temporal features from the last spatio-temporal block, which was able to extract the global correlations from multiple ECG signal segments.
- Experiment results on two public domain datasets of Apnea-ECG and UCDDB showed that the proposed CNN-BiGRU achieved a state-of-the-art performance, which outperforms the previous state-of-the-art methods. It means that the proposed CNN-BiGRU can be potentially deployed into a medical system to provide the SA monitoring service.

The remainder of this paper is organized as follows. Section 2 details the composition of the CNN-BiGRU model. In Sections 3 and 4 the results are presented and discussed. The conclusion is presented in Section 5.

## 2. Methods

### 2.1. Overview

**Figure 1:**
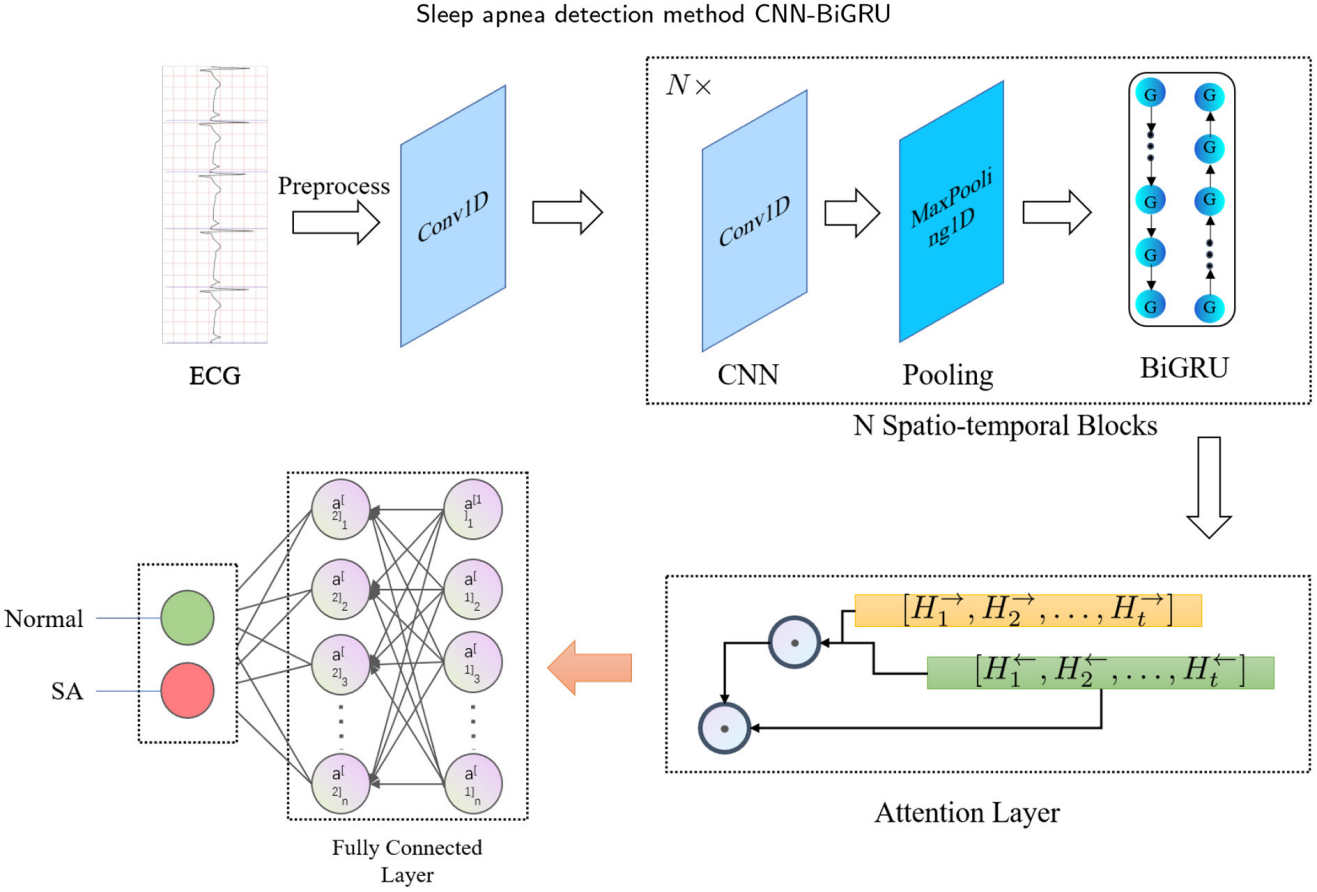
Flowchart of the proposed sleep apnea detection model.

The main idea of this study is to develop a fully automated (or end-to-end) spatio-temporal learning-based SA detection method, which is illustrated in Figure 1. First, in the pre-processing phase, the RR intervals and R-peak amplitudes are extracted from combining adjacent and labeled segments. Then, the RR intervals and R-peak amplitudes are fed into the proposed CNN-BiGRU model, which recycles through multiple spatio-temporal blocks to capture high-level spatio-temporal features. The whole method can be mathematically defined as follows: Given the input *X* ∈ ℝ^*D*^, a mapping function *f* : *X* → {0, 1} is learned, where *D* is the dimensionality of the input, and 0 and 1 denote normal and SA, respectively. Specifically, the spatio-temporal block consists of CNN, max-pooling and BiGRU layers. Furthermore, the attention mechanism is used to extract the most effective part of the spatio-temporal features and improve the accuracy. Finally, the fully connected layer is used to identify whether the labeled segment belongs to SA.

### 2.2. Preprocessing

**Figure 2:**
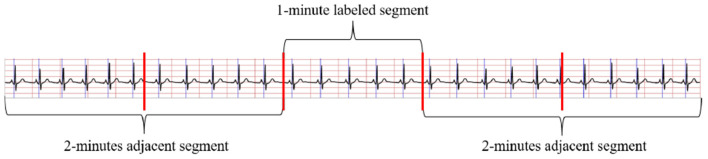
Five segments schematic diagram.

The method in Wang T. et al. (2019) to obtain the RR intervals and R-peak amplitudes were applied in this study to pre-process ECG signals. Considering adjacent segment information is useful for SA detection in each segment. As shown in Figure 2, both the labeled segment and its surrounding ECG signal ±2 segments (five segments total of 1 min) were extracted and processed. Firstly, the Hamilton algorithm (Hamilton, 2002) was used to find R-peaks and adjust the detection peak to match the local signal maximum. Then, RR intervals were calculated and the amplitudes were extracted using the locations of the R-peaks, while anomalous signals were removed. For physiologically unexplained points, median filters were chosen to solve the extracted RR intervals (Chen et al., 2015). The final problem of unequal time intervals between the RR intervals and amplitudes was tackled by cubic interpolation, which yielded 900 RR interval points and 900 amplitudes over 5 min segments.

### 2.3. The proposed CNN-BiGRU for SA recognition

The proposed CNN-BiGRU consists of spatio-temporal blocks of CNNs and BiGRUs, an attention layer and fully connections layers. These layers are introduced in detail as follows.

#### 2.3.1. Convolutional neural network

Convolutional neural networks are among the most common and efficient techniques that are widely used in various signal and image processing applications (Fan et al., 2018, 2019; Wang et al., 2018). A lightweight CNN model can be trained on a mixed-scale graph in order to extract deep features for the detection of obstructive SA (Mashrur et al., 2021). In this study, we used a one-dimensional CNN to extract spatial dependencies from ECG signals, which is mathematically defined as follows:


(1) $$\begin{array}{l} \left[\text{w}⊛\text{x}\right]\left(i\right)=\overset{L-1}{\underset{u=0}{\sum }}w_{u}x_{i+u} \end{array}$$

where **x**, **w**, *L* are the input, filter, and filter size, respectively.

The next layer of the CNN is generally the pooling layer. The max-pooling layer can be used to reduce mean value shift errors caused by bad initialization of weights (Wang L. et al., 2019). In this paper, the max-pooling layer was used to decrease the computational effort and to mitigate the overfitting problem by selecting the maximum value of each feature.

#### 2.3.2. Gated recurrent unit

Gated recurrent units (GRU) (Cho et al., 2014) represent a more advanced alternative to the simple recurrent neural network (RNN) and are more capable of learning long-term dependencies than vanilla RNN (Zhang et al., 2022). While both GRU and Long Short-Term Memory (LSTM) units have gating units that regulate the flow of information within the unit, GRUs do not have a separate memory unit, only update and reset gates. The *j*-th hidden unit of each mentioned gate is defined as follows:

Reset gates:


(2) $$\begin{array}{l} r_{j}=\sigma \left({\left[{\text{W}}_{r}\text{x}\right]}_{j}+{\left[{\text{U}}_{r}{\text{h}}_{\langle t-1\rangle }\right]}_{j}\right) \end{array}$$

where σ and [·]_*j*_ are the logistic sigmoid function and the *j*-th element of a vector, respectively; *t* is the time step, **x** denotes the input, and **h**_〈*t*−1〉_ represents the previous hidden state.

Update gates:


(3) $$\begin{array}{l} z_{j}=\sigma \left({\left[{\text{W}}_{z}\text{x}\right]}_{j}+{\left[{\text{U}}_{z}{\text{h}}_{\langle t-1\rangle }\right]}_{j}\right). \end{array}$$

Output:


(4) $$\begin{array}{l} h_{j}^{\langle t\rangle }=z_{j}h_{j}^{\langle t-1\rangle }+\left(1-z_{j}\right){\tilde{h}}_{j}^{\langle t\rangle } \end{array}$$

The weight matrices **W**_*r*_, **W**_*z*_, **W**_*x*_, **U**_*r*_, **U**_*z*_, and **U**_*x*_ are learned during training. The candidate hidden state ${\tilde{h}}_{j}^{\langle t\rangle }$ is computed as follows:


(5) $$\begin{array}{l} {\tilde{h}}_{j}^{\langle t\rangle }=\tanh\left({\left[{\text{W}}_{x}\text{x}\right]}_{j}+r_{j}\left[{\text{U}}_{x}{\text{h}}_{\langle t-1\rangle }\right]\right) \end{array}$$

The GRU can be heavily dependent on the dataset and the corresponding task, and the Apnea-ECG dataset works better with GRU than with LSTM, since it has fewer parameters and faster training (Chung et al., 2014). In this work, a bidirectional GRU was used to capture richer temporal information. By recursively computing the hidden states *H*_*t*_ in the forward and backward directions, the forward sequence *F* and the backward sequence *B* were obtained, respectively. This can be mathematically defined as follows:


(6) $$\begin{array}{l} H_{t}^{\to }=\left[h_{1}^{\langle t\rangle },h_{2}^{\langle t\rangle }\ldots h_{n}^{\langle t\rangle }\right] \end{array}$$

(7) $$H_{t}^{\leftarrow }=[h_{1}^{'\langle t\rangle },\;h_{2}^{'\langle t\rangle }\;\ldots h_{n}^{'\langle t\rangle }]$$

(8) $$\begin{array}{l} F={\left[H_{1}^{\to },H_{2}^{\to },\ldots ,H_{s}^{\to }\right]}^{T}\in {\mathbb{R}}^{s\times n} \end{array}$$

(9) $$\begin{array}{l} B={\left[H_{1}^{\leftarrow },H_{2}^{\leftarrow },\ldots ,H_{s}^{\leftarrow }\right]}^{T}\in {\mathbb{R}}^{s\times n} \end{array}$$

where *n* denotes the number of GRUs and *s* represents the total number of the time step.

#### 2.3.3. Attention mechanism

Dot-product attention (Luong et al., 2015) was used to extract the global correlation information from the input multiple ECG segments. Specifically, dot-production attention is applied on the forward sequence *F* and the backward sequence *B* of the BiGRU unit within the last spatio-temporal block. The attention score is calculated as follows:


(10) $$\begin{array}{l} \mathrm{Attention}\left(F,B,B\right)=\mathrm{softmax}\left(FB^{T}\right)B \end{array}$$

Using the attention mechanism allows the model to pay more attention to specific high-level spatial-temporal dependency information, improving the accuracy of the model. Note that dot-product attention is fast and spatially efficient because it enables a highly optimized code for matrix multiplication (Vaswani et al., 2017).

#### 2.3.4. Proposed CNN-BiGRU model

To better extract the spatio-temporal features of ECG signals, we have specially designed a SA detection model, named CNN-BiGRU. The proposed CNN-BiGRU model is mainly composed of a CNN layer, multiple stacked spatio-temporal learning blocks, an attention layer, and fully connected layers. First, a convolutional layer was used to extract the base features before using spatio-temporal blocks. A spatio-temporal learning block consists of a one-dimensional CNN, a max-pooling layer, and a BiGRU unit. The use of multiple spatio-temporal blocks enables the CNN-BiGRU model to extract high-level spatio-temporal features from the ECG signal. Specifically, our model is able to extract local spatial features of the R-peaks, as well as global temporal features of the heartbeat intervals. Then, the attention score of the fused high-level forward and backward features from the spatio-temporal blocks was calculated. This attention mechanism is able to further utilize the global spatio-temporal correlations from the multiple ECG segments. Finally, three dense layers were used for classification. Additionally, some of the layers were immediately followed by a dropout layer to mitigate the effects of overfitting. The mathematical expression of the whole model computation process is as follows: For the CNN input *X*, the output *C* is:


(11) $$\begin{array}{l} C=g\left(f\left(X;\text{W}\right)\right) \end{array}$$

where *g* denotes the ReLU activation function *g*(*x*) = max(0, *x*) and **W** denotes the convolution kernel.

As previously mentioned, this study uses BiGRU with the matrices **W**_*r*_, **U**_*r*_, **W**_*z*_, **U**_*z*_, **W**_*x*_, and **U**_*x*_ as the parameters to be learned. After reducing the size of the feature map through the max-pooling layer, the output *C* of the CNN was fed into the BiGRU. The output of the spatio-temporal block can be mathematically represented as follows:


(12) $$\begin{array}{l} F=\varphi \left(C;{\text{W}}_{r}^{\to },{\text{U}}_{r}^{\to },{\text{W}}_{z}^{\to },{\text{U}}_{z}^{\to },{\text{W}}_{x}^{\to },{\text{U}}_{x}^{\to }\right) \end{array}$$

(13) $$\begin{array}{l} B=\varphi \left(C;{\text{W}}_{r}^{\leftarrow },{\text{U}}_{r}^{\leftarrow },{\text{W}}_{z}^{\leftarrow },{\text{U}}_{z}^{\leftarrow },{\text{W}}_{x}^{\leftarrow },{\text{U}}_{x}^{\leftarrow }\right) \end{array}$$

where *F* and *B* are the stacked hidden states in the forward and backward directions, respectively. If the next layer of the BiGRU was a CNN, then *F* and *B* were concatenated along the channel dimension, otherwise the attention score *a* was calculated as follows:


(14) $$\begin{array}{l} a=\mathrm{softmax}\left(FB^{T}\right)B \end{array}$$

Finally, the attention score was entered into the fully connected layer for classification, and the labeled segments were classified to be SA or normal. Table 1 has listed the architecture details of the proposed CNN-BiGRU model. Specifically, the architecture contains three spatio-temporal blocks (see the layers of 2–5, 7–10, and 12–15, respectively in Table 1). And the dropout ratios in Table 1 have all been set to 0.2.

**Table 1:** Detailed parameter settings for the CNN-BiGRU model.

**Layer**	**Type**	**Number of** **filter/cell/unit**	**Kernel** **size**	**Activation** **function**
1	Convolutional	128	3	ReLU
2	Convolutional	128	3	ReLU
3	Max-Pooling	–	3	–
4	Dropout	–	–	–
5	Bidirectional GRU	128	–	Tanh
6	Dropout	–	–	–
7	Convolutional	128	3	ReLU
8	Max-Pooling	–	3	–
9	Dropout	–	–	–
10	Bidirectional GRU	128	–	Tanh
11	Dropout	–	–	–
12	Convolutional	128	3	ReLU
13	Max-Pooling	–	3	–
14	Dropout	–	–	–
15	Bidirectional GRU	128	–	Tanh
16	Attention	–	–	–
17	Flatten	–	–	–
18	Dense	64	–	ReLU
19	Dropout	–	–	–
20	Dense	64	–	ReLU
21	Dense	2	–	Softmax

### 2.4. Experimental settings

In order to enable an enhanced performance of the CNN-BiGRU model, the number of spatio-temporal blocks was tuned from 1 to 5. Adam optimizer (Kingma and Ba, 2017) and binary cross-entropy loss function were applied for parameter optimization. The learning rate and batch size were set to 0.001 and 128, respectively. The proposed model was trained for 40 epochs. In each training epoch, the model parameters were evaluated using the validation set and the best model parameters were saved to perform classification on the test set. Detailed training methods are described in Algorithm 1. Our model was implemented using the Tensorflow framework with a Tesla P100-PCIE-16GB graphics card.

**Algorithm 1:** CNN-BiGRU training.

**Input:** Given training set [*X, Y*] = {(*x*_1_, *y*_1_), (*x*_2_, *y*_2_), …, (*x*_*n*_, *y*_*n*_)} and validation set $\left[\tilde{X},Ỹ\right]$, the CNN-BiGRU model *f* with initialized parameters θ_0_, epochs *T*, learning rate α
**Output:** Trained model weight Θ
1: **Initialize** the parameters of the Adam optimizer: the exponential decay rates of the first and second order moments are estimated as *β*_1_ and *β*_2_
2: *m*_0_ = 0, *v*_0_ = 0
3: **for** *t* = 1 to *T* **do**
4: Forward-propagation: ŷ = *f*(*X*; θ_*t*_)
5: Compute loss error: $J\left(\theta \right)=-\frac{1}{n}\overset{n}{\underset{i=1}{\sum }}\left[y_{i}\ln\;\left({ŷ}_{i}\right)+\left(1-y_{i}\right)\ln\;\left(1-{ŷ}_{i}\right)\right]$
6: Compute the gradient of the current data: $g_{t}=\frac{\partial }{\partial \theta_{t}}J\left(\theta_{t}\right)$
7: Update network parameters by Adam optimizer: $$\begin{array}{l} m_{t}=\beta_{1}m_{t-1}+(1-\beta_{1})g_{t}\\ v_{t}=\beta_{2}v_{t-1}+(1-\beta_{2})g_{t}^{2}\\ \theta_{t}=\theta_{t-1}-\alpha \frac{\sqrt{\left(1-\beta_{2}^{t}\right)}m_{t}}{\left(1-\beta_{1}^{t})(\sqrt{v_{t}}+\epsilon \right)} \end{array}$$
8: **if** $f\left(\tilde{X};\theta_{t}\right)$ turns out better on the validation set than before **then**
9: Save weight θ_*t*_ to Θ
10: **end if**
11: **end for**

Various performance metrics, such as precision, specificity, *F*_1_ score, sensitivity, and accuracy, were used to assess the performance of the proposed model. These metrics are defined as follows:


(15) $$\begin{array}{l} \text{Precision}=\frac{TP}{TP+FP} \end{array}$$

(16) $$\begin{array}{l} \text{Specificity}=\frac{TN}{TN+FP} \end{array}$$

(17) $$\begin{array}{l} \text{Recall}=\frac{TP}{TP+FN} \end{array}$$

(18) $$\begin{array}{l} F_{1}\text{score}=\frac{2\times \text{Precision}\times \text{Recall}}{\text{Precision}+\text{Recall}} \end{array}$$

(19) $$\begin{array}{l} \text{Accuracy}=\frac{TP+TN}{TP+TN+FP+FN} \end{array}$$

where *FP*, *TP*, *FN*, and *TN* stand for “false positive,” “true positive,” “false negative,” and “true negative,” respectively. The SA class is the positive class in this study, while the normal class is the negative class.

This model was also evaluated using the receiver operating characteristic (ROC) and the related area under the curve (AUC).

## 3. Experimental results

### 3.1. Datasets

#### 3.1.1. PhysioNet Apnea-ECG dataset

**Figure 3:**
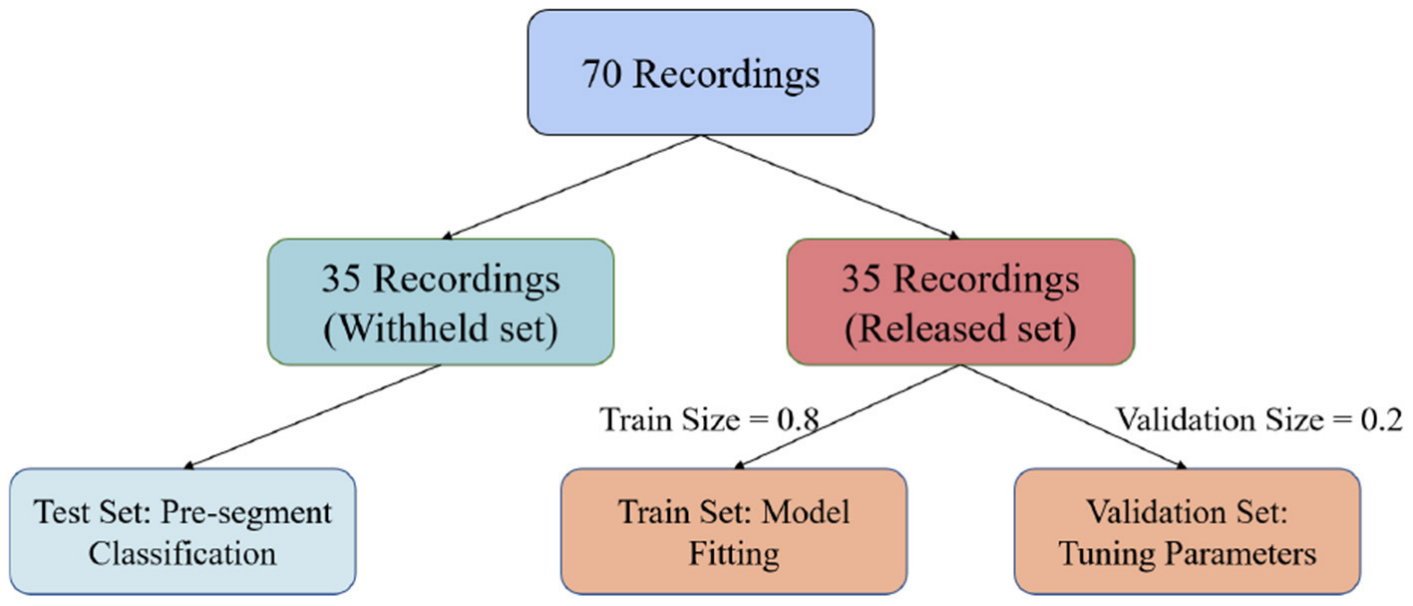
Datasets division methods on PhysioNet Apnea-ECG dataset.

In this paper, we used the PhysioNet Apnea-ECG dataset provided by Philipps University (Penzel et al., 2000) for model evaluation. The Apnea-ECG dataset has 70 recordings, including 35 records in the released dataset (a01–a20, b01–b05, and c01–c10) and 35 records in the withheld dataset (x01–x35). And the release set is used to train the model with the withheld set used to test the model. Regarding the released set, 20% of the 35 released data were used to validate the model and tune its hyperparameters (Figure 3). ECG recordings for this dataset were obtained from subjects with an AHI (apnea hypoventilation index) between 0 and 83. And these subjects ranged in age from 27 to 63 years and their body mass indices varied between 19.2 and 45.33*kg*/*m*^2^. The ECG signal was sampled at 100 Hz over a range of 401 to 587 min. The experts labeled each 1 min recording segments as SA or normal. According to our pre-proccessing method, the release and withheld set contained 17,045 and 17,302 segments, respectively. The experimental results show that CNN-BiGRU achieves an outstanding performance in SA detection.

#### 3.1.2. University college Dublin sleep Apnea database (UCDDB)

UCDDB was used as a second dataset to validate the performance of CNN-BiGRU. This database contains 25 full overnight polysomnograms from adult subjects suspected sleep-disordered breathing. ECG recordings of this dataset have been collected by a modified lead V2. We used ECG signals sampled at 128 Hz and with the durations ranging from 355 to 462 min. Following previous studies (Mostafa et al., 2017, 2020), we labeled a 1 min segment as SA if the segment contains more than 5 s of SA events. Considering the class imbalance problem of UCDDB, the data of patients without SA events (ucddb008, ucddb011, ucddb013, and ucddb018) are not used (John et al., 2021).

### 3.2. Classification performance on Apnea-ECG dataset

The SA detection involves two stages. The first stage is to detect whether a 1 min segment is SA. In the second stage, each patient is assessed for sleep quality overnight based on the results of the first stage.

#### 3.2.1. Per-segment classification on Apnea-ECG dataset

**Figure 4:**
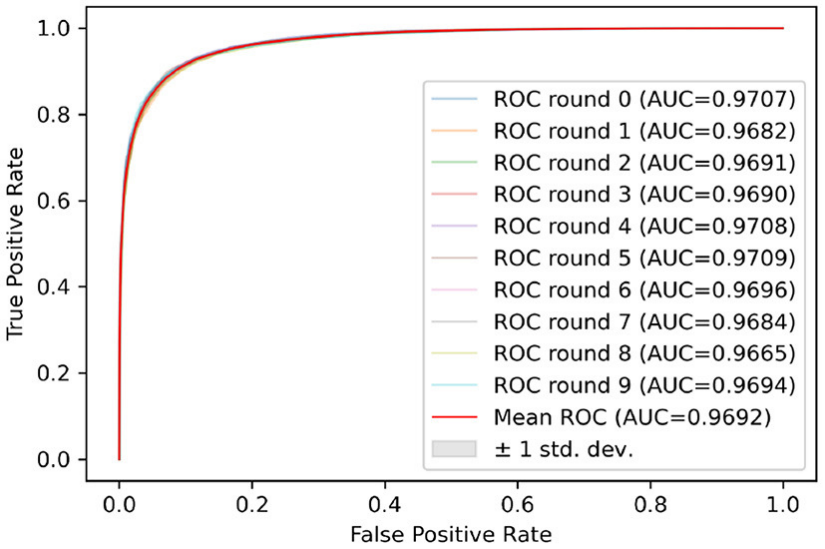
ROC curves for 10 random repeated runs on PhysioNet Apnea-ECG dataset.

Test sets were used to evaluate the effectiveness of the proposed model. First, the pre-processed 5 min ECG segments were fed into the CNN-BiGRU network to automatically extract the features. Then, the extracted features were fed into the fully connected layers, and the ECG signal of the middle segment was classified. The results of the CNN-BiGRU model with three spatio-temporal blocks for ten runs are listed in the Table 2. It is worth noting that the 10th experiment exceeded the average on all evaluation metrics. In addition, the 5th experiment reached the highest values of 91.54 and 88.82% for the accuracy and *F*_1_ score, respectively. To evaluate the classifier more comprehensively, Figure 4 shows the ROC curve and AUC. It can be seen that the model proposed in this study is stable, with an AUC value of 0.9692±0.0013.

**Table 2:** Per-minute detection performance Results on the Apnea-ECG dataset.

	**Accuracy (%)**	**Recall (%)**	**Specificity (%)**	**Precision (%)**	***F*_1_ score (%)**
1	91.25	85.69	94.71	90.96	88.24
2	90.80	86.84	93.26	88.88	87.85
3	91.18	88.10	93.09	88.79	88.45
4	91.14	88.37	92.86	88.49	88.43
5	91.54	87.72	93.92	89.95	88.82
6	90.95	86.38	93.79	89.62	87.97
7	91.18	84.84	95.12	91.52	88.05
8	91.36	83.78	96.07	92.97	88.13
9	91.37	86.41	94.45	90.63	88.47
10	91.41	86.64	94.38	90.53	88.54
Mean	91.22	86.48	94.16	90.23	88.30
Std	0.2098	1.360	0.9404	1.316	0.2833

Table 3 lists the comparison results between the CNN-BiGRU model and previous state-of-the-art works on the detection of per-minute ECG signals. Notice that the compared methods and the proposed models listed in Tables 3, 4 were all trained on the release set and evaluated on the withheld set. The results show that the average performance of CNN-BiGRU outperforms the previous optimal model in terms of accuracy, specificity, and *F*_1_ metrics. It is worth noting that CNN-BiGRU only underperforms the approach of literature (Li et al., 2018; Yang et al., 2022) in terms of the recall metric. However, our model achieved the *F*_1_ score of 88.3%, which is better than that of literature (Yang et al., 2022), while literature (Li et al., 2018) did not give *F*_1_ score.

**Table 3:** Per-minute detection performance comparison on Apnea-ECG dataset.

**References**	**Accuracy (%)**	**Recall (%)**	**Specificity (%)**	**Precision (%)**	***F*_1_ score (%)**
Bahrami and Forouzanfar (2021)	80.7	75.0	84.1	–	74.72
Sharma and Sharma (2016)	83.4	79.5	88.4	–	–
Li et al. (2018)	84.7	88.9	88.4	–	–
Feng et al. (2020)	85.1	86.2	84.4	77.2	81.4
Song et al. (2015)	86.2	82.6	88.4	–	–
Wang T. et al. (2019)	87.6	83.1	90.3	–	–
Sharan et al. (2020)	88.2	82.7	91.6	–	–
Yang et al. (2022)	90.3	87.6	91.9	–	87.3
Chen et al. (2021)	90.6	86.0	93.5	–	87.6
This work (mean)	91.2	86.5	94.2	90.2	88.3

**Table 4:** Per-recording detection comparison on Apnea-ECG dataset.

**References**	**Accuracy (%)**	**Recall (%)**	**Specificity (%)**	**MAE**	**Corr**
Song et al. (2015)	97.1	95.8	100	–	0.860
Sharma and Sharma (2016)	97.1	95.8	100	–	0.841
Li et al. (2018)	100	100	100	9.41	–
Wang T. et al. (2019)	97.1	100	91.7	–	0.943
Feng et al. (2020)	97.1	95.7	100	5.60	–
Shen et al. (2021)	100	100	100	4.23	–
Chen et al. (2021)	100	100	100	–	0.979
Yang et al. (2022)	100	100	100	2.70	0.985
This work	97.1	95.7	100	2.49	0.984

In summary, some previous works (Song et al., 2015; Sharma and Sharma, 2016) were based on feature engineering techniques that attempt to improve the performance by mapping high-dimensional training data to a low-dimensional feature space. However, this is time-consuming and ineffective. Deep learning methods can extract important features from ECG signals, and the DL-based methods (Chen et al., 2021; Yang et al., 2022) mentioned in Table 3 have all achieved good results, but their performance was inferior to that of the CNN-BiGRU model proposed in this paper. Our model uses spatio-temporal blocks, which can extract spatio-temporal features more effectively from ECG signals and provide better performance on SA classification.

#### 3.2.2. Per-recording classification on Apnea-ECG dataset

In order to further assess the quality of the subjects' sleep, an overall SA diagnosis of the subjects' recordings was performed. Each of the subjects' recordings consisted of multiple 1 min segments. The AHI is commonly used clinically as an indicator of whether a subject is suffering from SA. An individual is considered to have SA if the subject's AHI is >5 (Ruehland et al., 2009). The formula for calculating the AHI is as follows:


(20) $$\begin{array}{l} AHI=\frac{60\times N}{T} \end{array}$$

where *T* is the number of 1 min segments and *N* indicates the number of 1 min SA segments.

In the per-recording detection, the accuracy, sensitivity, specificity, and AUC of the CNN-BiGRU model were calculated on the retention set as 97.1, 95.7, 100, and 0.996%, respectively. The accuracy was 97.1% because the model misclassified one from the total 35 patients. More specifically, one subject (x12) with SA had an AHI of 6.75, whereas the proposed model calculated an AHI of 4.00, thus classifying the patient as normal. It is worth noting that the low precision per-segment approach may show better per-recording performance because of the relatively small amount of data in the test set (Wang T. et al., 2019). Therefore, according to the previous literature (Sharma and Sharma, 2016; Wu et al., 2021; Yang et al., 2022), the Pearson correlation coefficient (Corr) and mean absolute error (MAE) were also used as new evaluation indicators to ensure the reliability of the comparison. These metrics are defined as follows:


(21) $$\begin{array}{l} MAE=\frac{1}{N}\overset{N}{\underset{i=1}{\sum }}|AHI_{pre}^{i}-AHI_{\text{true}}^{i}| \end{array}$$

(22) $$Corr=\frac{\sum_{i=1}^{N}\left(AHI_{pre}^{i}-{\bar{AHI}}_{pre})(AHI_{true}^{i}-{\bar{AHI}}_{true}\right)}{\sqrt{\sum_{i=1}^{N}{\left(AHI_{pre}^{i}-{\bar{AHI}}_{pre}\right)}^{2}}\sqrt{\sum_{i=1}^{N}{\left(AHI_{true}^{i}-{\bar{AHI}}_{true}\right)}^{2}}}$$

where *N* is the number of recordings, and $AHI_{pre}^{i}$ and $AHI_{true}^{i}$ represent the predicted and true AHI values of the i-th recording, respectively.

Table 4 lists the comparison of the per-recording classification performance between the CNN-BiGRU model and state-of-the-art works in recent years. As mentioned above, traditional evaluation metrics do not provide a comprehensive and accurate assessment of model performance, and a better approach is to use MAE and Corr metrics. As listed in Table 4, our model achieved 2.49 and 0.984 for the MAE and Corr metrics, respectively. Our model achieved the best performance in terms of MAE metrics. On the Corr metric, literature (Yang et al., 2022) achieved the best value of 0.985, while our model achieved 0.984, which is a comparable result. Overall, our proposed model provides more competitive performance than those of the works presented in Table 4.

### 3.3. Classification performance on UCDDB dataset

Usually, UCDDB is utilized to evaluate the robustness of the SA detection models (Wang T. et al., 2019; Mashrur et al., 2021; Yang et al., 2022). Similarly, we evaluated our CNN-BiGRU model on UCDDB to demonstrate the model's robustness. Different from the Apnea-ECG dataset, UCDDB was not divided into the training set and test set by the original publishers. As a result, the previous works (Wang T. et al., 2019; Mashrur et al., 2021; Yang et al., 2022) had used their own splitting of training and testing sets in evaluations. In this paper, we used the same preprocessing method for the UCDDB as mentioned in Section 2.2. The difference is that the UCDDB is divided into a training set, a validation set and a test set with a proportion of 8:1:1. Due to the relatively small number of patients with SA at the UCDDB, the training set was balanced by oversampling the minority class (SA events). Meanwhile, per-recording testing is not performed for the same reason.

**Figure 5:**
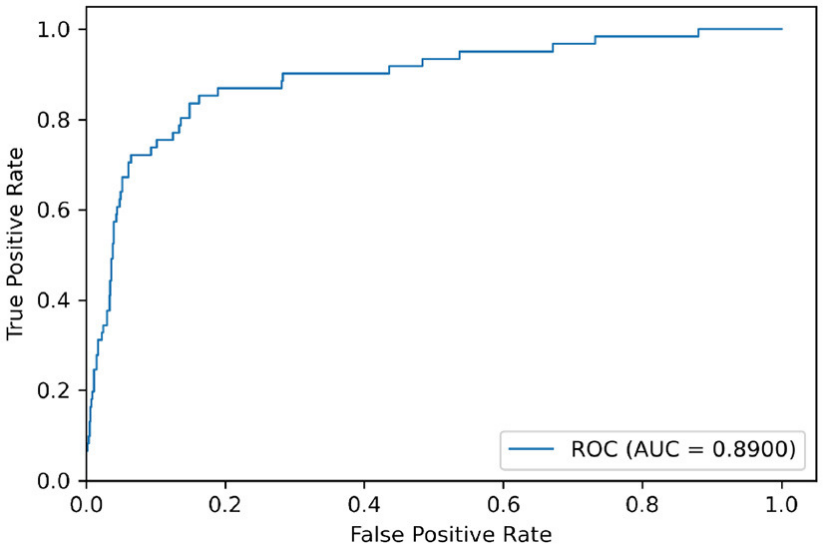
ROC curves on UCDDB when positive class is apnea.

We used the model trained on the Apnea-ECG dataset to continue training on the UCDDB training set, with the experimental settings mentioned in Section 2.4. On the UCDDB test set, the performance of the CNN-BiGRU model on the accuracy, recall, specificity, and AUC metrics reached 92.3, 70.5, 93.9, and 0.890%, respectively. Figure 5 shows the ROC curves and AUC of the proposed model for per-segment detection. Table 5 lists the results of CNN-BiGRU on the test set and compares them with other detection algorithms in the literature. The results show that the CNN-BiGRU model is far superior to the previous models, with an accuracy and specificity of 92.3 and 93.9%, respectively. In regard to recall metrics, we obtained a comparative result to the works (Mashrur et al., 2021). Compared to the Apnea-ECG dataset, our model has a significant decrease in the recall metric on the UCDDB. A major reason for this is that the ratio of pre-processed SA segments to all segments is about 1%, indicating that the class imbalance is intensified. Note that it is a rough comparison in Table 5, as there is no uniform data partitioning of training set and test set for UCDDB. In summary, our CNN-BiGRU model is useful for SA detection.

**Table 5:** Per-minute detection performance comparison on UCDDB dataset.

**References**	**Accuracy (%)**	**Recall (%)**	**Specificity (%)**	**Precision (%)**	***F*_1_ score**
Wang T. et al. (2019)	71.8	26.6	86.9	–	–
Papini et al. (2018)	74.7	50.6	84.0	–	–
Yang et al. (2022)	75.1	61.1	80.8	–	–
This work	92.3	70.5	93.9	46.7	76.0

## 4. Discussion

### 4.1. Hyperparameter tuning

**Figure 6:**
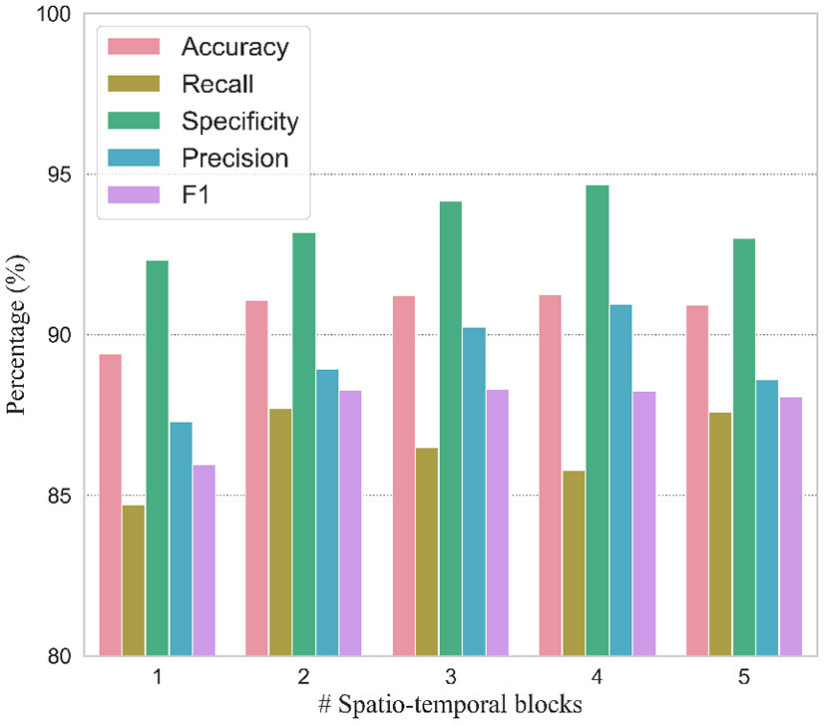
Hyperparameter tuning for the number of spatio-temporal blocks.

In order to verify the efficacy of spatio-temporal blocks, the number of spatio-temporal blocks was tuned from 1 to 5 on PhysioNet Apnea-ECG dataset. As shown in Figure 6 and Table 6, one spatio-temporal block model cannot effectively extract high-level spatio-temporal information. Meanwhile, too many spatio-temporal blocks also fail to learn high-level feature information due to the overfitting problem. Considering that Apnea-ECG dataset suffers from class imbalance, the *F*_1_ score became the main metric we considered. And the CNN-BiGRU model using three spatio-temporal blocks reached the highest values of 88.30% for *F*_1_ score. Therefore, we set the number of spatio-temporal blocks for CNN-BiGRU to 3 in this study.

**Table 6:** Comparison of per-segment detection results using different numbers of spatio-temporal blocks.

**# Spatio-temporal blocks**	**Accuracy (%)**	**Recall (%)**	**Specificity (%)**	**Precision (%)**	***F*_1_ score**
1	89.40 ± 0.4486	84.69 ± 1.867	92.32 ± 1.135	87.30 ± 1.509	85.95 ± 0.6510
2	91.08 ± 0.2314	87.70 ± 1.449	93.19 ± 0.9452	88.92 ± 1.262	88.28 ± 0.3309
3	91.22 ± 0.2098	86.48 ± 1.360	94.16 ± 0.9404	90.23 ± 1.316	88.30 ± 0.2833
4	91.26 ± 0.1953	85.77 ± 1.588	94.67 ± 0.8665	90.94 ± 1.243	88.26 ± 0.3714
5	90.92 ± 0.1502	87.59 ± 1.222	92.99 ± 0.7195	88.60 ± 0.9142	88.08 ± 0.2603

### 4.2. Ablation study

We conducted an ablation study on the Apnea-ECG dataset, considering that there was sufficient data in the Apnea-ECG dataset to fully evaluate the performance of the model. As shown previously, the CNN-BiGRU model uses a convolutional layer, spatio-temporal blocks, and an attention layer to extract features. Therefore, the results of the ablation experiments with the convolutional layer and the attention layer removed separately are listed in Table 7. It was found that removing either the convolutional layer or the attention layer will make the classification performance degrade. Specifically, the accuracy of the models with the convolutional layer removed and the attention layer removed is decreased by 0.47 and 0.67%, respectively. Overall, using the convolutional layer and attention layer improved the classification performance of the CNN-BiGRU model.

**Table 7:** Ablation of CNN-BiGRU.

**Convolutional** **layer**	**Attention** **layer**	**Accuracy (%)**	**Recall (%)**	**Specificity (%)**	**Precision (%)**	***F*_1_ score**
✓		90.55 ± 0.1128	85.28 ± 1.741	93.81 ± 1.183	89.60 ± 1.629	87.35 ± 0.2002
	✓	90.75 ± 0.2061	86.57 ± 2.054	93.35 ± 1.118	89.05 ± 1.468	87.76 ± 0.4489
✓	✓	91.22 ± 0.2098	86.48 ± 1.360	94.16 ± 0.9404	90.23 ± 1.316	88.30 ± 0.2833

### 4.3. Cross-dataset evaluation

Cross-dataset evaluations are performed to demonstrate the general performance of our proposed model, using the Apnea-ECG and UCDDB datasets. Specifically, the model is trained on one dataset and evaluated directly on another dataset. When CNN-BiGRU was trained on Apnea-ECG and tested on UCDDB, an accuracy of 85.3% and an F1-score of 50.5% were achieved. Similarly, when it was trained on UCDDB and evaluated on Apnea-ECG, the accuracy was 53.8% and the F1-score was 36.3%. It is found that the performances of cross-dataset evaluation are not satisfactory. To comprehensively understand the evaluation, we performed the same cross-dataset evaluation using a previous state-of-the-art model (Chen et al., 2021) listed in Table 3. The accuracy achieved was 85.9% and the F1-score was 51.1% using the UCDDB as the testing set and Apnea-ECG as the training set. They are very slightly better than those of our model (85.9 vs. 85.3%; 51.1 vs. 50.5%). However, when it was trained with UCDDB and tested on Apnea-ECG, the accuracy and the F1-score were 45.5 and 29.2%, respectively. Obviously, our model has outperformed this previous model (53.8 vs. 45.5%; 36.3 vs. 29.2%). In general, CNN-BiGRU is superior to the compared model (Chen et al., 2021), in terms of cross-dataset evaluation.

Finally, we attribute the low performance of cross-dataset evaluation to the following reasons: (1) the populations of datasets are different. For example, subjects with central apnea and periodic respiratory episodes are included in UCDDB; (2) the different sampling rates may impact the performance (the ECG signals are sampled at 100 Hz on Apnea-ECG while 128 Hz on UCDDB); (3) UCDDB has a severe class imbalance problem. In other words, the distributions of normal and SA are quite different between the two datasets.

## 5. Conclusion

In this study, a novel spatio-temporal learning-based model named CNN-BiGRU was explored to classify SA events from ECG signals. Specifically, the proposed CNN-BiGRU is an end-to-end deep learning model, which consists of multiple spatio-temporal blocks. Each block has the identical architecture with a CNN layer, a max-pooling layer, and a BiGRU layer. This architecture with repeated spatio-temporal blocks can well capture the morphological spatial feature information as well as the temporal feature information from ECG signals. Experiment results on the apnea-ECG dataset showed that the proposed CNN-BiGRU achieved an accuracy of 91.22 and 97.10% for per-minute classification and per-recording classification, respectively. And the accuracy on the UCDDB dataset reached 91.24%. In contrast to the previous state-of-the-art methods, our proposed CNN-BiGRU has an obvious advantage with a big margin. It means that the CNN-BiGRU can be potentially deployed into a medical system to help physicians to screen out SA patients to avoid malignant events. In future work, we will further apply the proposed model to real healthcare systems.

## Data availability statement

Publicly available datasets were analyzed in this study. This data can be found at: PhysioNet Apnea-ECG: https://physionet.org/content/apnea-ecg/1.0.0/; UCDDB: https://archive.physionet.org/physiobank/database/ucddb/.

## Author contributions

Conceptualization: WZ and WM. Methodology: JC and MS. Software: JC. Writing: WZ and JC. Project administration: WZ. All authors contributed to the article and approved the submitted version.

## Funding

This work was partly supported by the National Key R&D Program of China under Grant 2019YFB1804003.

## Conflict of interest

The authors declare that the research was conducted in the absence of any commercial or financial relationships that could be construed as a potential conflict of interest.

## Publisher's note

All claims expressed in this article are solely those of the authors and do not necessarily represent those of their affiliated organizations, or those of the publisher, the editors and the reviewers. Any product that may be evaluated in this article, or claim that may be made by its manufacturer, is not guaranteed or endorsed by the publisher.
